# Action sound–shape congruencies explain sound symbolism

**DOI:** 10.1038/s41598-020-69528-4

**Published:** 2020-07-29

**Authors:** Konstantina Margiotoudi, Friedemann Pulvermüller

**Affiliations:** 1 Brain Language Laboratory, Department of Philosophy and Humanities, WE4, Freie Universität Berlin, 14195 Berlin, Germany; 2 Berlin School of Mind and Brain, Humboldt Universität zu Berlin, 10099 Berlin, Germany; 3 Einstein Center for Neurosciences, Berlin, 10117 Berlin, Germany; 4 Cluster of Excellence “Matters of Activity”, Humboldt Universität zu Berlin, 10099 Berlin, Germany

**Keywords:** Psychology, Human behaviour, Neuroscience, Anthropology

## Abstract

Sound symbolism, the surprising semantic relationship between meaningless pseudowords (e.g., ‘maluma’, ‘takete’) and abstract (round vs. sharp) shapes, is a hitherto unexplained human-specific knowledge domain. Here we explore whether abstract sound symbolic links can be explained by those between the sounds and shapes of bodily actions. To this end, we asked human subjects to match pseudowords with abstract shapes and, in a different experimental block, the sounds of actions with the shapes of the trajectories of the actions causing these same sounds. Crucially, both conditions were also crossed. Our findings reveal concordant matching in the sound symbolic and action domains, and, importantly, significant correlations between them. We conclude that the sound symbolic knowledge interlinking speech sounds and abstract shapes is explained by audiovisual information immanent to action experience along with acoustic similarities between speech and action sounds. These results demonstrate a fundamental role of action knowledge for abstract sound symbolism, which may have been key to human symbol-manipulation ability.

## Introduction

Sound symbolism is an umbrella term that covers the non-arbitrary associations between meaningless speech sounds and sensory or other meanings^1^ (for a review see^2^). The iconic links between pseudowords and abstract visual shapes is the most popular demonstration of this phenomenon. In the present study, the term “sound symbolism” will refer to these latter associations. In his seminal book entitled “Gestalt Psychology”, Köhler^3^ described the classic “maluma–takete” paradigm in which humans match a round figure to a ‘round’ sounding pseudoword, such as “maluma”, and a sharp figure to a ‘sharp’ sounding pseudoword such as “takete”, thus presupposing an abstract ‘resemblance’ between the otherwise meaningless symbol (pseudoword) and the corresponding shape, possibly based on shared modality general abstract properties. Many experimental studies confirmed Köhler’s example and demonstrated the postulated iconic speech-sound/meaning mappings across languages^4–6^, even at early age (for a meta-analysis see^7^) and across stimulus modalities^8,9^. Furthermore, the ability to perform well on sound symbolic tasks has been related to word learning capacity in young children^10–12^.

These results led to some skepticism towards the linguistic Saussurean^13^ position that the relationship between form and meaning of signs is arbitrary and even suggest an important role of sound symbolic mechanisms in language development^14^ and evolution^15^. Specifically, vocal iconic mappings between infants’ first spoken words and the referents these words are used to speak about appear to be substantial, so that iconic signs may have a special status for our ability to talk about things not present in the environment, a feature sometimes called ‘displacement in communication’^6^. Today, iconicity and sound symbolism along with their bootstrapping role in language development and evolution are widely upon agreement^15^, with recent evidence coming from a study in great apes showing the human specificity of sound symbolic mappings. Margiotoudi et al.^16^ tested humans and great apes in the same two-alternative forced choice (2AFC) task. Both species were presented with different ‘round’ versus ‘sharp’ sounding pseudowords and were required to select a (round vs. sharp) shape that best matched the pseudoword. Humans but not great apes showed significant congruency effects. These results suggest that, similar to language, sound symbolism is a human-specific trait. It has also been argued that sound symbolism may depend on human-specific neuroanatomical connectivity also relevant for language^17,18^, in particular on the presence of strong long-distance connection between frontal and temporal perisylvian areas^16^.

Despite the numerous studies documenting sound symbolism, few theories attempt to explain the underlying mechanism. Sound symbolism may be considered as a specific type of crossmodal correspondence implicating the matching of shared sensory or semantic features across modalities^19^. In this spirit, the frequency code theory proposed by Ohala^20^ states that the association of large (small) objects with segments of low (high) frequency, such as vowels having low (high) second formant (i.e., /o/ vs. /i/) is due to the statistical co-occurrence of these features in nature. For instance, large (small) animals vocalize in low (high) frequencies due to differences in the size of their vocal apparatuses; large animals have large vocal apparatuses resulting in the production of lower frequencies compared to smaller animals. However, whereas this explanatory scheme applies nicely to phonetic-acoustic correspondences, to small versus large shapes, it is not immediately clear why sharp and round shapes should tend to co-occur with certain phonemes and articulations. Therefore, this approach seems to be too limited to provide a full account of sound symbolic effects. A related perspective puts that crossmodal links between acoustic and visual information may be based on the amount of energy across modalities, and therefore on a ‘more or less’ in sensory neuronal activation^21^. Whereas this position seems well-suited to provide a candidate account for the correspondences of ‘vivid’ and ‘flat’ speech sounds and colors^22,23^, it would need to be shown how an explanation of the mapping of round abstract figures on the pseudoword “maluma” and one of spiky stars and edges on “takete” could be marshalled along these lines. Therefore, also this approach seems to be too limited to provide a full account of sound symbolic effects.

An eminent and highly cited theory addressing the mechanism of sound symbolism specifically, also highlighting its putative importance for the emergence of protolanguages in language evolution, is that of Ramachandran and Hubbard^24^. The authors propose a “syneasthetic articulatory account” of “maluma–takete” type of associations between meaningless pseudowords and abstract visual forms. In their “bouba-kiki” example, the authors explain that the sharp edges of a spiky shape mimic the sharp phonemic inflections and the sharp movement trajectory of the tongue on the palate when uttering the pseudoword “kiki”. Hence, the principal idea is that there are non-arbitrary mappings between features of tongue movement trajectories which characterize the articulatory act and lead to the production of characteristic speech sounds. Ramachandran and Hubbard propose that these spatial characteristics and acoustic effects of the articulatory act provide the glue essential for sound symbolic iconic knowledge and that this knowledge became the basis for the emergence of protolanguages and for linking spoken signals to referent objects. However, as to the best of our knowledge, there is no strong experimental evidence supporting this synaesthetic articulatory model.

Ramachandran and Hubbard’s proposal can be criticized on theoretical and empirical grounds. The knowledge most crucial for bridging between visual and speech modalities, that about the movement trajectory of the tongue, is part of procedural knowledge and therefore not necessarily and easily accessible to the cognizing individual^25^. Decades of phonetic research were necessary to document articulatory trajectories, first with x-ray and later- on with electromagnetic articulography^26,27^, to find out about the complex and sometimes surprising moves and turns of different articulators in speech production^28–30^. A simple abstract shape, such as a spiky star, appears as a quite distant approximation of such complexity. Unfortunately, the most important articulator, the tongue, is hidden in the mouth and therefore not visible to speakers or listeners. Making the visual features of these movements the key component of the explanation of sound symbolism may therefore appear as questionable from a theoretical perspective. Until now, a systematic comparison of articulatory trajectory features characterizing the production of pseudoword forms such as “takete–maluma” and the abstract shapes these spoken items respectively relate to according to sound symbolic experiments is still missing, so that it remains unclear whether this model can account for the range of phonetic contrasts leading subjects toward selection preferences for sharp and round shapes.

**Figure 1:**
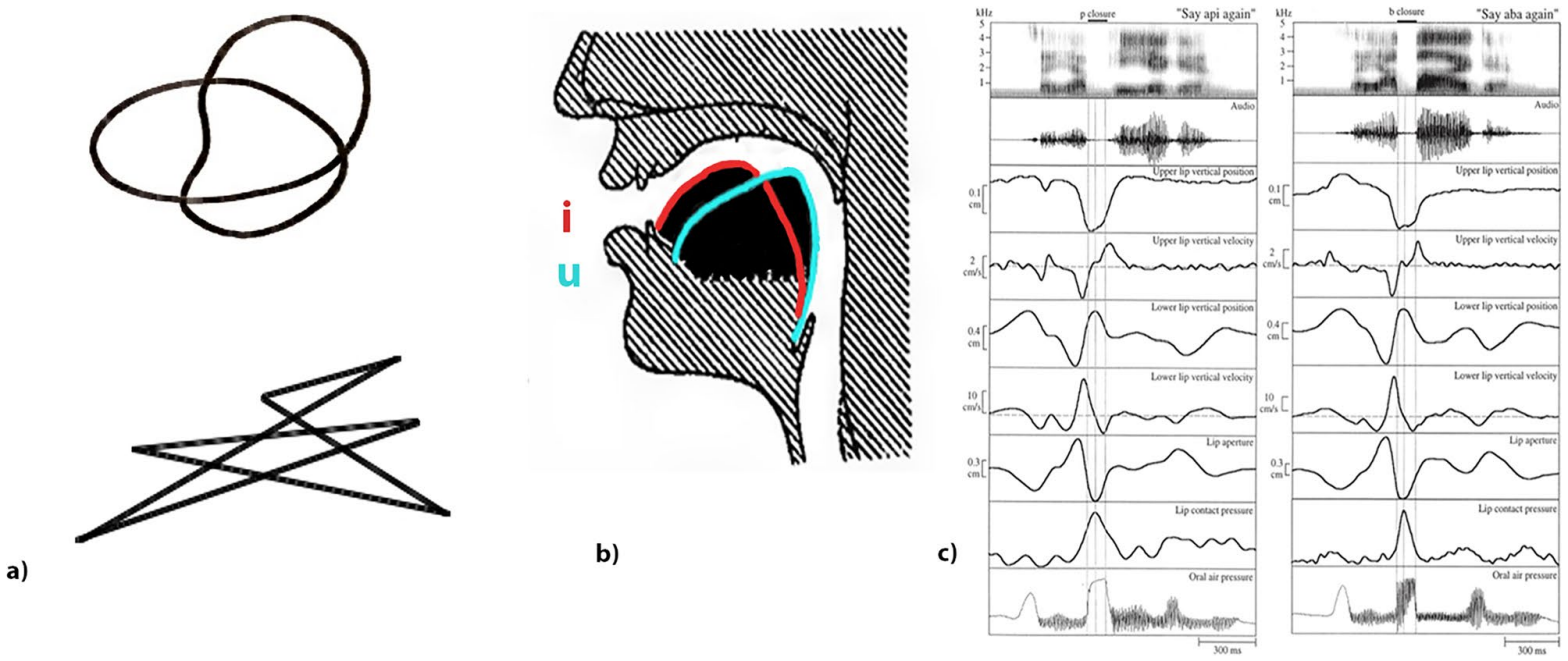
(**a**) Köhler’s original stimuli “maluma–takete”. The upper shape corresponds to the pseudoword “maluma” and the lower to “takete”. Reproduced from Köhler^3^. (**b**) Tongue positions of the vowels /i/ (in red) and /u/ (in turquoise). The shape of the tongue for the vowel /i/ does not resemble the edgy “takete” figure depicted at Köhler’s work. Adapted from Jones^34^. (**c**) Movements/velocities of lips during the production of the pseudowords “api” (left panels) and “aba” (right panels). Note the absence of any similarity between movement trajectories and ‘sharp’ shapes [such as the lower item in panel (**a**)]. Reprinted by^35^ with permission.

Furthermore, experimental evidence can be marshalled against the most established explanation attempt for sound symbolism: it is well known that dark and light vowels, such as /u/ versus /i/ lean toward ‘sharp’ versus ‘round’ interpretations^10,31^ although these are not associated with clear movement trajectory contrasts that could motivate such sound symbolic links. As shown in Fig. 1a,b the shapes of the classic “maluma–takete” example show little resemblance to the shapes of the tongue position of a typical ‘sharp’ sound, /i/, or that of a typical ‘round’ one, /u/. Both tongue shapes look very similar to each other and differ only with respect to the (backness) position (high at the front vs. back) of the anterior part of the tongue, without showing different sharpness versus roundness features for the two vowels. Likewise, when looking at lip trajectories recorded with articulography during the production of syllables such as /pi/ versus /ba/, which again lie on opposite sides of the round-sharpness continuum, the movements appear equally smooth (see Fig. 1c). These examples seem incompatible with the idea of similarities between the ‘round-’ and ‘sharpness’ of speech sounds on the one hand and articulator shapes or trajectories on the other; thus, they argue against the proposed articulatory account of sound symbolism.

Whereas the tongue and a range of other important articulators are hidden in the mouth, other body parts are clearly visible to the acting individual. Particularly hand movements, are clearly visible to the person performing them and to any interacting partners. When learning to move and, later on, to perform complex goal directed actions, the information about how to perform an act and the perceptual aspect, how the movement is carried out and how the gestures look and sound like, go together and can be associated in a Hebbian learning process (for discussion, see^32,33^). As a result, sensorimotor representations develop in the brain. Computer simulations of learning in cortex indicate that these multimodal representations are carried by distributed and connected groups of neurons interlinking action and perception knowledge, so-called action perception circuits. These multimodal neuronal devices can provide a basis of crossmodal information exchange and for the computation of the shape of a movement trajectory based on the motor schema or vice versa. We here explore the possibility, that these action perception circuits for hand actions provide the mechanistic basis of sound symbolic associations. If this is the case, we would not only expect that human subjects show corresponding abilities (a) to detect sound symbolic congruencies and (b) to match hand action sounds to the visual forms resulting from action trajectories, but we would also expect these abilities to be correlated across individuals, so that experts in sound symbolism would also be excellent sensorimotor action mappers and vice versa, whereas individuals less skilled in one of the tasks should also perform not-so-well on the other. This leads to the *primary hypothesis*, that there is a significant correlation between subjects’ ability to perform sound symbolic mappings and their performance on solving sound–shape mapping tasks for hand actions. In particular, any such correlation should be significantly stronger than any correlation between the performances on the sound symbolic task and a control condition closely matched to the latter, which, in our present case, was the 2AFC. The new model would also postulate that sound symbolic mappings are a by-product of action mappings, due to analogies and physical correspondences between the acoustic features of action sounds and speech and similarities between typical sound symbolic shapes and the shapes resulting from action trajectories. This latter postulate implies further important secondary predictions: that there are further significant correlations between subjects’ abilities to map information about actions and sound symbolic entities across modalities and domains, that is, between action sounds and abstract visual shapes and, furthermore, between maluma–takete-like pseudowords and the shapes of hand action trajectory shapes.

To test these novel predictions, we performed using the same 2AFC paradigm, (1) the classic sound symbolic (or *SoSy*) “maluma–takete” experiment along with 3 others. (2) A hand *Action* condition examined the matching of visual and acoustic aspects of pen drawing, whereby the sounds of the pen moving on the paper when drawing elementary visual shapes led to the acoustic stimuli and the corresponding visual items were the visual shapes, produced by moving the pen. In both, conditions (1) and (2), half of the stimuli were round and the other half sharp. The remaining two conditions resulted from crossing of the former two, so that (3) hand action-produced visual stimuli had to be selected for sound symbolic pseudowords (*Crossed1 condition*) and (4) sound symbolic abstract shapes (or the “maluma–takete” type shapes) for hand action sounds (*Crossed2 condition*). As a further condition, a control 2AFC task was administrated with animal pictures and the sounds the depicted animals typically produce, so as to probe general sensorimotor knowledge unrelated to shape-sound correspondences intrinsic to human-specific actions. The Animal task was administrated to obtain an estimate of performance with variations in 2AFC task performance, evaluating general attentional, motor or perceptual skills across the test population. At the end of the experiment, an additional paper-and-pencil attention test (6) was administrated to control for variability in the subjects’ performance level on a sustained attention task. We predicted that, if action knowledge links underlie the sound symbolic mapping of auditory to visual features and vice versa, specific significant correlations across all action and sound symbolic tasks would emerge, that is, across conditions (1)–(4), but not between tasks (1)–(4) and any of the control tasks (5) or (6).

## Methods

### Subjects

Twenty-four right-handed adults (20 females, age M = 25.04, SD = 3.47) participated in the study. The subjects were native speakers of different languages (8 German, 3 Turkish, 2 Mandarin, 2 English, 2 Greek, 2 Arabic, 1 Spanish, 1 Italian, 1 Albanian, 1 Cantonese, 1 Hungarian). To assure that all subjects understood the oral instructions given in English, all participants succesfully completed the online Cambridge Assessment English test for the English language prior to the experiment^36^. In order to be eligible for the study, subjects had to have on the aforementioned test a score equal to or above the B1 level in English. All subjects had normal hearing and normal or corrected to normal vision. One subject could not complete the experiment due to health issues and her data was therefore excluded from the analysis. Subjects were recruited by way of written announcements at the Freie Universität Berlin. All methods of the study were approved by the Ethics Committee of the Charité Universitätsmedizin, Campus Benjamin Franklin, Berlin and were performed in accordance with relevant guidelines and regulations to the Declaration of Helsinki. All subjects provided written informed consent prior to the participation to the study and received 20 euros for their participation.

### Stimuli

**Figure 2:**
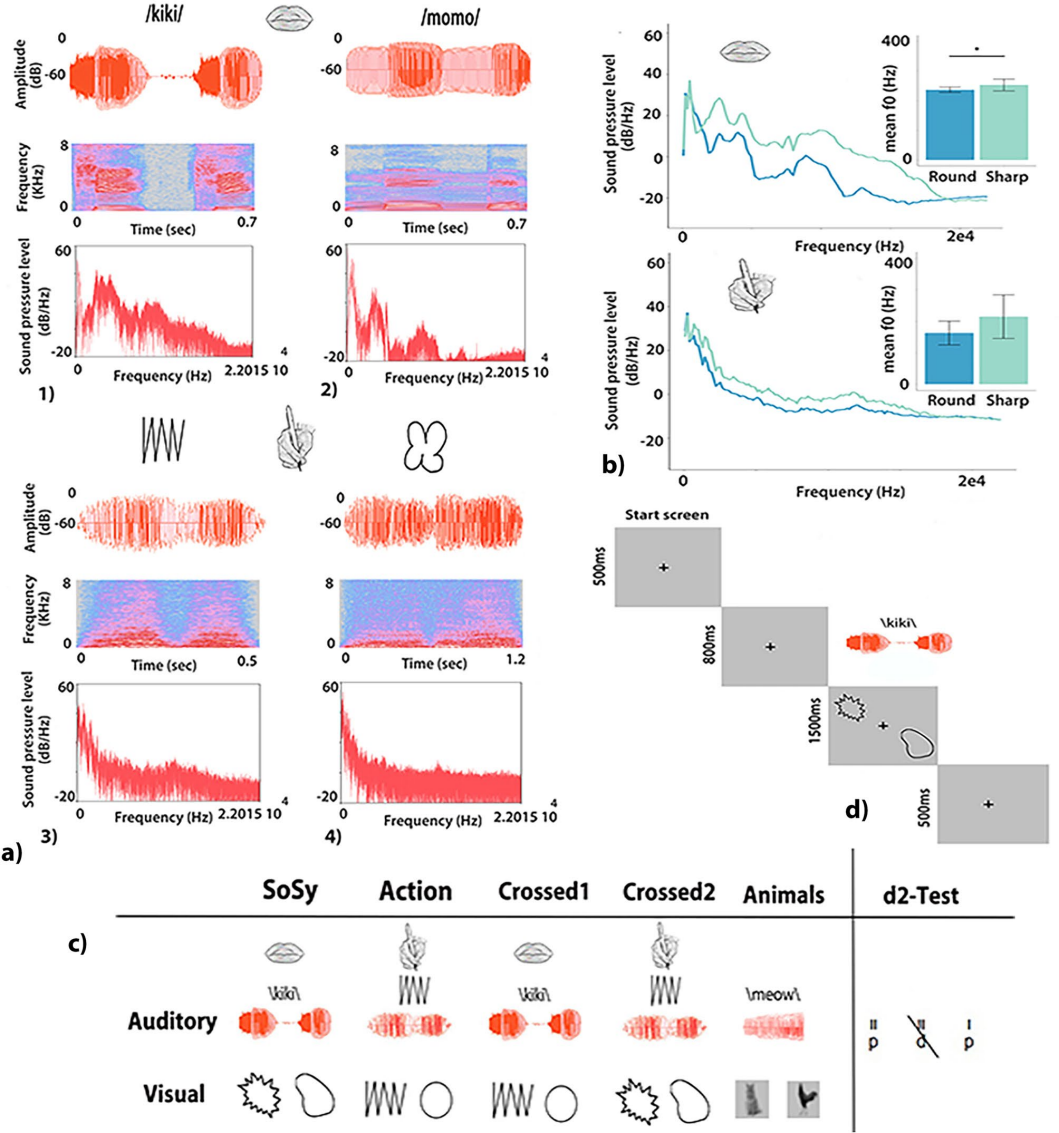
(**a**) Waveforms, spectograms and power spectral densities (PSD) of SS_pwd_ (top panels) and Action_snd_ (bottom panels), (1) “kiki”, a ‘sharp’ rated dissylabic SS_pwd_, (2) “momo”, a ‘round’ rated dissylabic SS_pwd_, (3) a ‘sharp’ and (4) a ‘round’ sounding Action_snd_. (**b**) Average PSD for both ‘sharp ’and ‘round’ sounding SS_pwds_ (top panel) and Action_snds_ (bottom panel), segmented in 145 bins. Mann–Whitney-U-tests were used to calculate the difference of PSD average values between round and sharp categories. For both SS_pwd_ (W = 14,919, $p<0.001$) and Action_snd_ (W = 7,526, $p<0.001$) there was a significant difference of PSD values between ‘sharp’ and ‘round’ sounding stimuli. Bar plots show average and standard deviations of fundamental frequencies (F0) for ‘sharp’ and ‘round’ sounding categories. Mann–Whitney-U-tests were revealed a significant difference only for the SS_pwds_ (W = 17, $p<0.01$) between ‘sharp’ and ‘round’ sounding categories and not for the Action_snds_ (W = 5, $p=0.15$) for the F0 measure. (**c**) The table summarizes the combination of auditory and visual stimuli for the five forced choice tasks. The sixth column depicts an example from the d2 attention task as presented in the paper–pencil version. (**d**) Schematic representation of the experimental procedure for the SoSy condition. The procedure was the same for all the forced choice tasks with modifications on presentations time depending on the type of the stimulus.

We included the following stimulus types:

- Sound symbolic abstract shapes (SS_sh_): Twenty shapes, all of them similar to shapes commonly used in experiments on sound symbolism, were selected from^16^ (see Supplementary Material Table S1), 10 sharp and 10 round ones. However, whereas filled versions had previously been used, we here followed Köhler’s original strategy using black-on-grey (RGB 0,0,0 vs. RGB 192,192,192) line drawings (size: $350 \times 350$ pixels). This was done to achieve similarity to the action shapes (see below).
- Sound symbolic pseudowords (SS_pwd_): Twenty bisyllabic SS_pwd_ previously used and described in the study of Margiotoudi et al. (for the list of pseudowords see Supplementary Material Table S1). These included items typically used in sound symbolic experiments, such as “kiki” and “momo”. We adopted 10 ‘sharp’ and 10 ‘round’ sounding SS_pwd_. All recordings were saved at 44.1 kHz sampling rate with an average duration of all SS_pwd_ $\mathrm {m}= 578 \pm 41.28$ ms.
- Action shapes (Action_sh_): Action shapes were generated by drawing a selection of abstract shapes. We focused on elementary geometric shapes, such as circle, oval, sine wave and triangle, saw tooth, plus slightly more complex figures including two of the elementary shapes, e.g. small circle/triangle embedded in a larger one, figure-of-eight/hourglass figure. We selected the 10 shapes whose corresponding sounds had previously been rated the 5 best ‘round’ and ‘sharp’ sounding ones. A further rating (N = 13, by subjects recruited online via mailing lists) ascertained that the 10 action shapes selected were also among either the 5 most ‘sharp’ (M = 1.30, SD = 0.47) or the 5 most ‘round’ rated ones (M = 6.53, SD = 0.32); the ratings of these stimulus grounds significantly different from each other (W = 25, $p=0.01$) as revealed by a Wilcoxon signed rank test (see Supplementary Material Table S1).
- Action sounds (Action_snd_): A pen producing a clearly audible (but not uncomfortable) sound was used to generate sounds while drawing the abstract shapes of the action shape condition described above. Recordings were taken in a sound-proof booth, using a stereo built-in X/Y microphones Zoom H4n Handy Recorder (Zoom Corporation, Tokyo, Japan) saved at 44.1 kHz. For rating the action sounds, a separate group of subjects (N = 41, recruited online via mailing lists) judged the ‘sharpness’ or ‘roundness’ of each hand drawing recording on a 7-point Likert scale, ranging from 1-completely ‘sharp’ to 7-completely ‘round’. We selected the 5 action sound recordings receiving the highest ‘sharp’ ratings (M = 2.18, SD = 0.23) and the 5 ‘round’ ones (M = 5.23, SD = 0.40). These corresponded to the shapes selected for the action shape category described above. The rating scores obtained for these two subgroups of action sounds were significantly different from each other (W = 25, $p=0.007$), as revealed again by a Wilcoxon-signed rank test. The Action_snd_ were edited to make them acoustically comparable to the bisyllabic SS_pwd_, which all consisted of two syllables. To this end, we restricted the length of each action sound so that it included only the first two acoustic maxima and therefore resembled a bisyllabic speech item (see Fig. 2a,b). Moreover we applied fade in and out functions for the first and the last 100 ms so as to remove any on-and offset artifacts. The average duration of action sounds was $\mathrm {m}= 934 \pm 473.19\,{\hbox {ms}}$.
- Animal pictures: Twenty pictures of common animals, two for each animal species, were selected. As preliminary testing showed ceiling performance on the animal-picture-sound matching task, animal pictures were slightly blurred to introduce a level of difficulty in the task and require subjects to be attentive.
- Animal sounds: Finally, we chose ten different common sounds produced by the well known animals whose pictures were selected for the task control condition. Each animal sound had a duration of 300 ms.

All auditory stimuli were normalised for sound energy by matching their root mean square (RMS) power to 24.0 dB and they were edited using the programs Audacity (2.0.3) (Free Software Foundation, Boston, USA) and Praat (Institute of Phonetic Sciences, University of Amsterdam, the Netherlands). The visual stimuli were edited on Adobe Photoshop CS5.1 (Adobe Systems Incorporated, San Jose, CA, USA).

### Design and procedure

The experiment was programmed in E-Prime 2.0.8.90 software (Psychology Software Tools, Inc., Pittsburg, PA, USA). Subjects performed a 2AFC task with five different conditions. During the 2AFC task, subjects are presented with a sound/pseudoword, followed by two alternatives (pictures/shapes) and they have to make a forced choice on which picture/shape is the target stimulus that best matches to the preceding sound. In the first four conditions, we explored any congruency effects between the different sound symbolic and hand action related visual and auditory stimuli. Specifically, in the first condition (sound symbolic, SoSy) subjects had to match SS_pwd_ to SS_sh_. In the second condition (Action) they had to match Action_snd_ to Action_sh_ stimuli. For the third and fourth conditions, we crossed the auditory and visual stimuli of the previous two conditions. Hence for the Crossed1 condition we used the SS_pwd_ with Action_sh_ and for the Crossed2 condition the Action_snd_ with the SS_sh_. Condition five, the Animal task, was introduced for any effects (e.g. attention, perception, motor responses) induced by the 2AFC task itself that could affect the performance of the subjects generally. Finally, the last paper-and-pencil d2-attention test was introduced in order to control for variable levels of sustained attention for each subject (see Fig. 2c).

In all five alternative forced choice conditions, each trial started with the presentation of a fixation cross for 500 ms followed by the presentation of an auditory stimulus ‘the prime’. Due to the different nature of the sounds (SS_pwd_, Action_snd_, Animal sounds ) presentation time of these prime stimuli were either 800 ms (SS_pwd_) or 1,700 ms (Action_snd_), or for 300 ms (Animal sounds). Next, the two shapes, always one sharp and one round, appeared diagonally on the screen, one on the upper left, the other on the bottom right or in the other two corners. One of these visual stimuli was the target matching with the previous prime sound. During the fifth condition, two animal pictures were presented with only one of them matching to the preceding animal sound. The two visual stimuli stayed on screen for 1,500 ms (SS_pwd_ /Action_sh_). Presentation time was shortened to 1,000 ms (Animal picture) so as to slightly challenge the subjects in the otherwise too easy Animal task. Responses were collected while visual stimuli were on screen. Every trial ended with the presentation of a blank slide lasting for 500 ms (see Fig. 2d). All visual stimuli were presented on a grey background (RGB 192,192,192). Each condition consisted of 160 trials. Half blocks of 80 trials were separated by a pause screen. The subjects decided when to resume the next half block. Within each condition, trials were randomized; the combinations of auditory and visual stimuli were unique in each half block.

In a sound proof and dimly lit room, subjects sat in front of a 23 in. LCD monitor (screen refresh rate 75 Hz; screen resolution $1{,}280 \times 1{,}024$). The auditory stimuli were presented via two Logitech speakers (Model NO: Z130) (Logitech Europe S.A., Lausanne, Switzerland) located at each side of the screen. Responses were recorded via two buttons on a Serial Response Box (SRBox, Psychology Software Tools, Inc, Pittsburg, PA, USA). Before the initiation of the experiment and at the beginning of every new condition, subjects received on the screen the following written instructions: “During the experiment, two pictures will appear, one low and one high on your screen, presented after a sound. Please choose one of the two pictures that best matches the sound you just heard”. No specific instructions were given to the participants regarding speed or accuracy. Button presses had to be given with the index and middle fingers of the right hand. The up/down button was used for selecting the visual stimuli appearing at the corresponding side of the screen. After completing the computer experiment, all subjects completed in English the d2 cancellation test^37^. The d2 paper–pencil test is a psychometric measure of sustained attention. During the test, takers are asked to discriminate between different visual stimuli, and cross out the target stimuli (the letter d” with two dashes). The d2-test procedure lasted about 5 min.

### Data analysis

All analyses were performed on the analysis tool R (version 3.4.3, R Developement Core Team)^38^. Trials with reaction times greater than the time response window or without button-press were excluded. All variables were checked for normality using the Shapiro–Wilk normality test. To check if subjects’ selection of shapes was influenced by the preceded sound, we performed Wilcoxon signed-rank tests to compare the number of congruent responses against chance level for every condition separately after controlling for multiple testing with Bonferroni correction (adjusted threshold $p=0.05/5=0.01$). Moreover, we compared the congruency performance between the four conditions with a Kruskal–Wallis test with pairwise multiple comparison adjusted using Bonferroni correction. In order to explore whether the congruency detection performance of the subjects in a given AFC condition was correlated with their congruency detection performance with the other AFC conditions and with their performance on the d2 test, we performed a number of correlations. Specifically, we calculated Spearman’s correlation coefficients to assess pairwise linear relationships for the number of congruent responses of each subject between AFC conditions, and between each AFC condition and the scores acquired from the d2 test. From the d2 test, we calculated the concentration performance (CP) score, which is the number of correctly crossed-out items minus the errors of commission. CP scores can provide an index of sustained attention and takes into account both speed and accuracy of the performance. The higher the CP score the higher the attention of the subject. A false discovery rate correction (FDR,^39^ threshold set at 0.05) controlled for multiple comparisons using the p.adjust function in R. Furthermore, for comparing the size of the correlation coefficients among the sound symbolic, action and crossed conditions and between with the control AFC task, we performed 12 multiple comparisons with Steiger’s Z one-tailed tests on these coefficients. All *p* values were adjusted with Bonferroni correction for multiple testing ($p=0.05/12=0.004$).

In order to check, whether performance in the first four conditions was further influenced by other variables, we fitted generalized linear mixed models (GLMM) with a binomial error structure using the package lme4^40^. The dependent variable was congruency, that is, whether the selected shape matched the shape corresponding to the primed sound. We included SS_pwd_/Action_snd_ (‘sharp’ vs.‘round’) and trial number as fixed effects. We used a maximal random effect structure with random intercepts for subject, SS_pwd_ or Action_snd_ and for the combinations of the presented shapes and random slopes for each trial nested within these random effects. We used the likelihood ratio test (LRT) to check if the predictor variables improved the fit of the model; these were calculated by comparing the full model to a reduced model that included all terms except for the fixed effect term in question. Chi-square and *p* values were computed using the function drop1 from the R package lme4.

## Results

**Figure 3:**
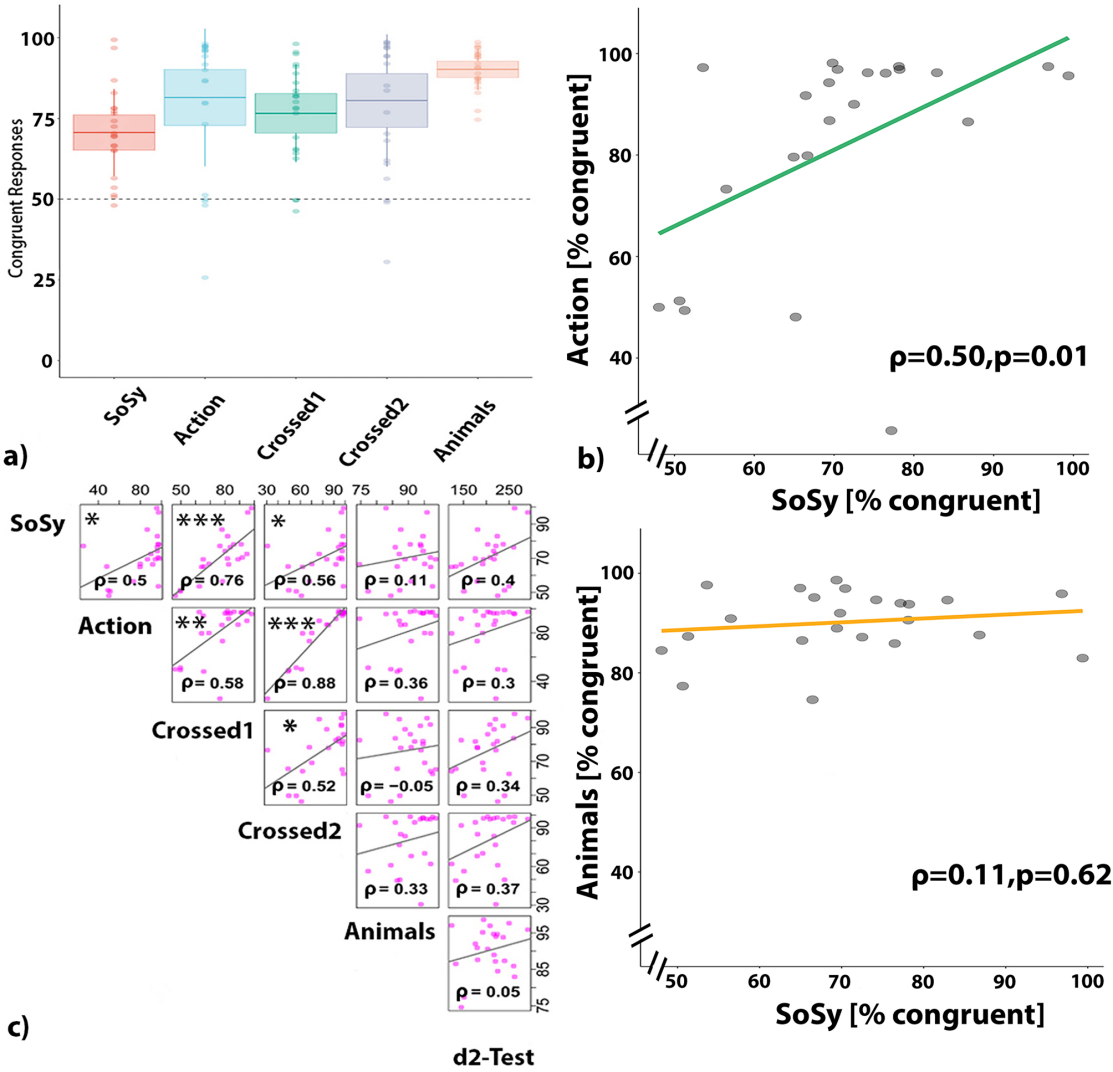
(**a**) Percentages of congruent responses for the two-alternative forced choice conditions, the SoSy (red), the Action (blue), the Crossed1 (green) and Crossed2 (purple) and the Animals tasks (orange). For the first four conditions, congruency is quantified as the proportion of times each individual matched a ‘sharp’ sounding SS_pwd_/Action_snd_ to a sharp shape or a ‘round’ sounding SS_pwd_/Action_snd_ to a round shape. For the Animal task, congruency means correct matching of sound and selected animal picture. Light colored circles show the percentage of congruent responses for each individual. Boxplots show standard deviations, lines show means and the whiskers show 95% confidence intervals (CIs). The dashed line at 50% shows chance-level performance. (**b**) Bivariate scatterplots with regression lines and correlation coefficients ($$\rho$$ values) of Spearman correlations between SoSy and Actions (green), and between SoSy and Animal task (yellow). (**c**) Bivariate scatterplots with regression lines and correlation coefficients ($$\rho$$ values) of Spearman correlations calculated across congruency scores of subjects obtained for all possible condition pairs, including the five alternative forced choice conditions and the concentration performance (CP) scores of the d2-test. Significant correlations after FDR correction (threshold set at: 0.05) are marked with asterisks ($$*{p}<0.05;**{p}<0.01;***{p}<0.001$$).

Across all five conditions, a total of 5.8% of trials were excluded from the analyses because of null or long-delay responses. Shapiro’s–Wilk tests, performed on the percentage of congruent responses obtained from each subject for each of the five conditions, revealed that normality was violated for two conditions, (Action: W = 0.75, $p<0.001$) and for (Crossed2: W = 0.83, $p<0.001$) and hence non-parametric statistics were performed. In each condition, subjects showed above chance performance on congruency detection between the presented sound and the selected pictures. In particular, for SoSy, subjects performed above chance (V = 273; $p=0.001$) with an average 70.64% congruent responses. Similarly, above chance performance was observed for the Action condition with an equally strong congruency bias of 81.50% congruent responses (V = 244, $p=0.001$). Comparable results were obtained for the two crossed conditions, Crossed1 and Crossed2 with 76.59% of congruency (V = 270, $p=0.001$) and 80.56% (V = 266, $p=0.001$) congruent responses. The Animal task yielded 90.20% congruent responses (V = 276, $p=0.001$) (see Fig. 3a). In addition, the Kruskal–Wallis test showed a statistically significant difference between congruency performance levels across the first four conditions ($\chi ^{2}(3)=8.45, p=0.04$). However, none of the pairwise differences survived Bonferroni correction ($p<0.012,=0.05/4=0.012$).

Next, we addressed the *primary hypothesis* whether the roundness and sharpness classifications of sounds were related to each other across the SoSy and the Action conditions. Spearman rank correlations revealed a significant positive correlation between subject specific congruency percentages obtained from the SoSy and the Action conditions ($\rho =0.50, p =0.01$ before and $p=0.03$ after FDR correction). Notably, correlations of SoSy task performance with that on the closely matched 2AFC control task failed to reach significance (see Fig. 3b). One may argue that the significant correlation between Action and SoSy conditions and its absence in the comparison between SoSy and 2AFC control task may just reflect a threshold effect. To address this possibility, the Steiger’s Z test was used to assess any significant differences between correlation coefficients. Using this test, the crucial correlation of SoSy and Action condition performances (SoSy vs. Action) was significantly greater (Steiger’s Z = 1.67, $p=0.04$) than that between SoSy and 2AFC control task results.

To address the secondary hypotheses, that the mapping between SoSy and Action conditions was in part due to similarities in acoustic and visual stimuli used across these tasks, we calculated all pairwise correlations between the SoSy, Action and Crossed conditions (FDR corrected). The highest positive correlations were observed between Action and Crossed2 conditions ($\rho =0.88$, $p=0.001$), followed by SoSy and Crossed1 ($\rho =0.76$, $p=0.001$). These condition pairs both share the same sounds: the Action and Crossed2 conditions the action sounds and the sound symbolic and Crossed1 conditions the pseudowords. Therefore, the correlations indicate that subjects generalized very well across shape types: they performed similarly on matching SS_sh_ and Action_sh_ to the same sounds. This implies a degree of similarity between SS_sh_ and Action_sh_, which is obvious, as the same visual elements resulting from elementary round and edgy movements where the components of these shapes. Clearly significant, although slightly less impressively than the former, were the correlations between conditions that shared the same shapes, i.e. Action_sh_ or SS_sh_. Action_sh_ were similarly well matched to Action_snd_ as to SS_pwd_ ($\rho \,=\,0.58, p=0.01$), and the same applied for the SS_sh_ ($\rho =0.56, p=0.02$). These results indicate a similarity in processing the different sound types, of actions and speech sounds, a topic to which we will return in discussion below. Moreover, a positive correlation was also observed between the two crossed conditions, Crossed1 and Crossed2 ($\rho \,=0.52, p=0.03$) (see Fig. 3c).

Remarkably, there was not a single reliable correlation between the closely matched action-unrelated 2AFC task using animal pictures and sounds and any of the four experimental conditions addressing SoSy and Action related knowledge (see Fig. 3c). Likewise, the secondary control task, d2 test performance using the CP score index, failed to yield any significant correlations with any of the sound symbolic or action related conditions. Also, performance on the two control tasks was uncorrelated. The absence of correlations with any of the two control tasks shows that the performance variability of our subjects in sound symbolic and action related conditions was not related to attention or to the cognitive and motor demands of the forced choice task.

To address possible threshold effects related to the secondary hypothesis, the Steiger Z-test was used once again, now to more systematically compare all possible pairings of correlation coefficients across SoSy-Action domains on the one hand—the ‘within domain correlations’—and correlations between these and the task-control condition on the other—‘between-domain correlations’. The 12 tests performed between ‘within’ and ‘between domain’ correlations revealed 8 significantly different correlation coefficients ($p<0.05$), and even after most conservative Bonferroni correction (corrected critical $p=0.05/12=0.0042$), five of these remained significant (for details, see Supplementary Materials Table S2). This is evidence for the specificity of correlations across action- and sound-symbolic domains.

The predictor variable of SS_pwd_ type significantly improved the model for SoSy condition ($\chi ^{2}$(1) = 12.72, $p=0.001$) with subjects having more congruent responses for ‘round’ sounding SS_pwd_ than ‘sharp’ ones, a finding previously reported by Margiotoudi et al.^16^, which may indicate a ‘roundness bias’ in the matching choices of pseudowords in sound-symbolic experimental context. This effect was, however, not seen in other conditions. The factor SS_pwd_/Action_snd_ type did not improve any of the other models with Action not reaching significance ($\chi ^{2}(1)=3.26,p=0.07$), not either in the conditions Crossed1 ($\chi ^{2}(1)=1.7,p =0.18$), or Crossed2 ($\chi ^{2}(1)=0.96, p=0.32$). Therefore, any roundeness bias was not present in the crossed conditions sharing either SS_pwd_ or SS_sh_ stimuli with the SoSy condition. As a result, the roundness bias specifically observed in the SoSy condition cannot be driven by the pseudoword or shape stimuli shared between SoSy and Crossed conditions.

## Discussion

In the present study, we used several two-alternative forced choice, 2AFC, and control tasks to investigate the role of action knowledge in sound symbolism, i.e. the human specific ability to detect abstract iconic correspondences. We replicated the well-known classic “maluma–takete” effect in the sound symbolic, or SoSy, condition and found similar and statistically even more impressive result for an Action condition, where subjects had to match abstract shapes drawn with a pen and the sounds produced by drawing them. Notably, by crossing both conditions and thus pairing action shapes with pseudowords (Crossed1) and abstract shapes with action sounds (Crossed2), we also found that our experimental subjects consistently judged sound symbolic correspondences, across SoSy and Action stimuli, thus classifying some shapes and sounds coherently as either round and others as sharp.

However, subjects performed differently well on such classification and we therefore asked, whether their differing levels of ability to interlink meaningless speech with abstract symbolic shapes might be systematically related to their performance on associating the shape of hand movements with the sounds produced when performing such movements. Surprisingly, when correlating the subjects’ performance on the sound symbolic and the action task, there was a significant correlation, which even exceeded that found between the 2AFC control task unrelated to sound symbolic or action knowledge. Furthermore, when investigating all four sound symbolic, action and crossed tasks, we consistently found significant correlations across these. No significant correlations were found between sound symbolic or action related conditions and the main control tasks examining general performance on the 2AFC control task and sustained attention abilities.

These results, demonstrate that human subjects’ sound symbolic ability to associate meaningless speech with abstract shapes is intrinsically related to their knowledge about the sounds of bodily actions performed with the hand and the shapes of the trajectories of such movements. We submit that this knowledge about sound symbolic relationship in our experimental subjects is best explained by associative learning between manual actions and the observed shapes and sounds they produce, along with visual similarities between action and sound symbolic shapes as well as by acoustic similarities between action sounds and speech.

One may argue that articulatory sounds and their related articulatory trajectories may provide an alternative explanation for the sound-symbolic capacity of humans, as previously stated by Ramachandran & Hubbard (henceforth R&H)^24^. These authors stated that “[...] the sharp changes in visual direction of the lines in the [takete] figure mimics the sharp phonemic inflections of the sound kiki, as well as the sharp inflection of the tongue on the palate.” (ref.^24^, p. 19). Therefore, the postulate is about (1) a correspondence between ‘sharp’ visual line arrangements and ‘sharp’ sounds and about (2) a correspondence between ‘sharp’ visual arrangement and ‘sharp’ inflections of the tongue. Statement (1) appears to us rather metaphorical. The word ‘sharp’ means different things in the context of visual shapes and sounds. Any ‘similarity’ needs explanation, but cannot be taken for granted and used to provide an explanation. The crucial question is why we perceive ‘sharp’ shapes and sounds as somewhat similar, and this question remains unanswered by R&H’s statement. Whereas their first statement does not provide an explanation, R&H’s postulate (2) comes with an empirical implication: that visual shapes of the abstract figures must in someway or another, resemble the “inflections of the tongue on the palate”. Unfortunately, the authors do not provide empirical or experimental evidence. Meanwhile, a body of data is available addressing this issue. So is there in fact resemblance between sharp and edgy figures and sharp tongue or articulator inflections on one side and rounding figures and round and smooth articulator movements?

As mentioned in the Introduction above, knowledge about the trajectories of our articulations is implicit and procedural so that one may dispute conscious access to it. As most articulators and their trajectories are not visible to the speakers or interlocutors, it may therefore be asked how any knowledge about these trajectories could come in into play in the cognitive task of sound symbolic matching. Decades of phonological and phonetic research were necessary to uncover these articulatory trajectories, so that it appears as a little optimistic to assume that the relevant knowledge is freely available as a basis for explicit sound-symbolic decisions. If we focus on articulators that are visible, as for example the lips, only a limited fraction of relevant features can be covered. But even worse: as we will elaborate below, there seems to be a lack of evidence for resemblances between abstract shapes and the shapes of articulators or articulatory trajectories while uttering phonemes that contribute to the perception of a pseudoword as either ‘sharp’ or ‘round’-sounding.

The actual movement trajectories revealed by articulatory phonetic research do not seem to exhibit edges, but, instead, appear as similarly smooth and round for round-sounding and sharp-sounding phonemes. In the case of consonants, even the most ‘spiky’ examples, such as /p/, are produced by similarly smooth lip movements as the ‘round’ sounding /b/ (see Fig 1c). Rather than being based on sharp and round articulatory movements, their acoustic differences relate rather to the precise timing of articulatory movements or the level of oral air pressure released^35,41^. Turning to vowels, one may want to point to examples such as /i/ and /u/—where one phoneme is ‘round’ from a sound symbolic perspective and from a phonetic perspective too (the /u/)—as it requires lip rounding, whereas the other one is sound-symbolically ‘sharp’ and not-rounded phonetically (the /i/). However, in spite of the existence of such matches, mismatching counterexamples are easy to find. Items that are uniformly classified as ‘rounded’ from a phonetic perspective, such as /y/ and /u/—since both require lip rounding—end up at different ends of the sound-symbolic roundness-sharpness continuum (see for /y/^42^ and for /u/^43^). Sharp-sounding but phonetically ‘rounded’ /y/ violates the correspondence as do round-sounding but phonetically not-rounded /$\alpha$/ and /a/^43,44^. Therefore, lip rounding as a phonetic feature is not a reliable indicator of sound symbolic categorization.

The unreliable status of articulatory movements as indicators of sound symbolic properties is further confirmed when observing the trajectories of articulators hidden in the mouth. The tongue shape and trajectory while articulating the vowel /i/, a high front vowel producing a strong bias towards ‘sharp’ sound-symbolic judgements, does not show features of a spiky figure, nor would the ‘round’ sounding /u/ and /o/ exhibit any smoother tongue trajectories (for details, see tongue trajectories of vowels in^27^). This phoneme, /i/ is produced with the tongue close to the roof of the palate, thus creating a large cavity at the back of the mouth, which does not mirror a sharp structure nor is edgier compared to the tongue shape characteristic of /u/, which has the back of the tongue close to the palate (see Fig. 1b). Similarly for the sharp versus round sounding syllables /pi/ and /ba/, we explained above that the movements of the articulators do not have corresponding ‘round’ versus ‘sharp’ features (see Fig. 1c). Studies which mapped tongue movements online, for example with articulatory tractography, found comparable trajectories, for example of the back of the tongue, for different vowels^27^. Therefore, it appears that the envisaged ‘similarity’ of articulatory movements to sharp and round shapes cannot be used as an explanatory basis of sound symbolism.

Although the similarity between articulatory movements and round versus sharp shapes cannot account for the general phenomenon of sound symbolism, we do not wish to exclude that an acoustic-articulatory speech component might contribute in some way to such an explanation. In contrast, however, the shared roundness and sharpness features of overt hand movements shared across the visual shapes of their trajectories and the sounds of these actions are well supported by our current data and generally applicable to various speech sounds. Therefore, they offer a perspective on explaining sound symbolism.

Given that correlations across experimental subjects’ performance were observed, one may argue that any significant effects may be due to general between-subject differences, such as, for example, differences in arousal, sustained or visual attention, or swiftness and skill in solving computerized tasks requiring button presses. As one possibility, it could have been the relatively greater level of attention of individual subjects to sounds and figures along with their acoustic and visual details that co-determined comparatively better performance on both sound-symbolic and action alternative forced choice tasks. To explore these possibilities, two control tasks were administrated. The first task was designed to closely match the 2AFC task frequently used in sound symbolic experiments, but for the control task, no sound symbolic or action related information was involved. Subjects had to match animal pictures to sounds produced by animals, a task not drawing upon information about human action. Note that this task did not only control for possible differences in attention levels but likewise for putative variability in perceptual or motor skills (e.g., slow vs. fast responders). We consider this AFC task the main control condition, as it most closely controlled for various features of the critical tasks. Furthermore, a second control task was administered, the d2-test, which provides an estimate of levels of sustained attention. Interestingly, whereas all correlations of performance across the four sound symbolic/action related conditions achieved significance, (at least at a level of significance uncorrected for multiple comparisons), all correlations between one of the latter and a control task were insignificant. Note that the large number of tests made it necessary to control for multiple comparisons, and, as mentioned in results, even after most rigorous correction a relevant number of tests were still significant, thus providing strong evidence for the proposed action-based explanation. However, the primary hypothesis of our current study addressed one and only one correlation, that between SoSy and Action matching tasks (and thus did not call for multiple comparison correction). As this correlation was significant and, crucially, proved significantly stronger across subjects than that between the sound symbolic and the main (2AFC) control task, we can conclude that the primary hypothesis, that sound symbolic and sensorimotor action mappings are intrinsically related, receives strong support. Our results also show that the SoSy-Action correlation we observed across individuals is not explained by perceptual, task-performance-related or general cognitive differences between experimental subjects.

The significant correlations in the crossed conditions together with those between SoSy and Action condition indicate that some acoustic features are shared between the ‘round’ sounding SS_pwd_ and Action_snd_ produced in creating roundish hand movements and lines tracing them and likewise for the ‘sharp’ category. As the correlation between crossed and SoSy/Action conditions that shared their acoustic stimuli—either SS_pwd_ or the Action_snd_ drawings—led to the most impressive results, with $\rho$ values ranging around 0.8, it appears that these visual stimuli differing between these condition pairs resembled each other. This was doubtlessly the case, because the two visual shape categories, that is sound symbolic and elementary action shapes, shared edges/spikes or smooth curves. Based on these visual similarities, performance correlations between conditions sharing acoustic stimuli can easily be explained.

Likewise, for the conditions sharing the visual shapes, there were significant results. This indicates that, also across the acoustic stimuli, the SS_pwd_ and Action_snd_, there was a degree of similarity. Looking at individual stimuli, this hypothesis can be supported. Figure 2a,b show acoustic wave forms, spectograms and frequency composition of sound stimuli (from the SS_pwd_ and Action_snd_ categories) commonly judged as ‘sharp’ or ‘round’. It can be seen that in both, the ‘sharp’ Action_snd_ and SS_pwd_ have brief breaks or sudden pronounced sound energy drops between the two maxima of the sound, whereas, the ‘round’ sounding stimuli lack such an abrupt break or substantial dip. Also, the ‘sharp’ items typically exhibit relatively more power in the high frequency range, which is either absent or much reduced for the ‘round’ items; instead the latter include relatively more energy at the lower frequencies (see average power spectra in the bottom diagrams in Fig. 2b). These observations were supported by statistical analyses. We found significantly different overall spectral power for both ‘sharp’ Action_snd_ (W = 7,526, $p<0.001$) and SS_pwd_ (W = 14,919, $p<0.001$) as compared with their respective ‘round’ categories. In addition, the first peak of the Fourrier spectrum was found at significantly lower frequency for ‘round’ stimuli than for ‘sharp’ ones for the SS_pwd_ (round: 236.9 vs. sharp: 252.8 Hz $p<0.01$). Similar patterns were revealed for the Action_snd_ (round: 162.7 vs. sharp: 214.7 Hz $p>0.05$), although the differences did not reach significance in this case, maybe due to the limited number of actions (five per category).

In summary, our results revealed a reliable correlation between our subjects’ performance on the classic task of sound symbolism and an action condition. This finding is best explained by the similarities between stimulus categories, in particular between sound-symbolic shapes and the drawn shapes on the one hand and between the pseudowords and the sounds resulting from shape production on the other. The correlation suggests that, due to these physical similarities, similar mechanisms are at work in the processing of actions and sound symbolism.

These results offer a novel explanation of sound symbolism. As the link between abstract shapes and meaningless speech is difficult to explain, similarities between these shapes and the correlation between the trajectories and sounds of hand actions can easily be learned when observing oneself or another person drawing or otherwise producing such shapes. Hence, it is possible to explain sound symbolic knowledge as a consequence of action knowledge, i.e. the learnt correspondences between the shapes and consequent sounds of hand movement.

It is worth mentioning that previous studies have already shown that, beyond sound–shape associations, round and sharp dynamic body movements can also be associated to “maluma” versus “takete” pseudowords^9^ as well as to certain speech sounds^8^. Shinohara et al.^8^ reported that front vowels and obstruents are more likely to be associated to sharp than round dynamic gestures and demonstrate a further fact of abstract cross-modal sound symbolism. In this study, the takete–maluma-type sound symbolism is considered just one type of sound symbolism and the movement-phonemic links represent a different one, so that all of these cross-modal links are instantiations of “a general feature of our cognition”. These findings, although providing great evidence for the link between actions and round or sharp sounding speech sounds, do not address whether action knowledge may be the basis of abstract sound symbolic knowledge. In addition, the actual sounds created by executing these body movements were not investigated. Here, in contrast to Shinohara et al., we propose that there are not different types of sound-symbolic knowledge—e.g., for static figures and for actions—but that one type (action knowledge) explains the other seemingly ‘abstract’ types by experience-based associative learning and physical similarity, rather than by pre-established abstract links.

One may object that the visual and acoustic stimuli used in this experiment were too limited to fully support such general conclusion. Other visual shapes, for example more complex ones than the elementary ones used in this study, may show other relevant features not explored here. However, we believe that these possible caveats do not generally invalidate our argument. If other, for example more complex shapes allow for additional sound–shape associations, this does not invalidate the links obvious from our present stimuli using elementary figures. Other ways of producing sounds—for example produced by ‘drawing’ shapes with a sword in the air, or the tip of the foot in the sand—will certainly produce different sounds. Nevertheless, it seems plausible that the acoustic physical features varying between a sharp and round on-paper drawing are similar to the features emerging from the same shapes being drawn with sword or foot. In fact, we have experimented with different ways of producing action trajectories and sounds and finally selected the pen-on-paper strategy because it led to stimuli that were easy audible and easy to control for a range of acoustic properties (see Methods). Although we have not investigated this systematically, our data indicate that acoustic and visual features differences are shared across different ways of action production. Therefore, these differing features may provide the cues for visual-acoustic binding of information essential in sound symbolic knowledge.

The knowledge about an action together with its visual and auditory aspects must be stored in the cortex by a memory trace. Such traces may be local neuron circuits localized in a specific part of the brain devoted to semantics, a so-called ‘semantic hub’^45^. However, this type of model does not explain the knowledge link between memory mechanism and the perceptual and action-related knowledge it needs to connect with (grounding). Therefore, grounded memory models propose distributed neuron circuits as the carriers of memory^46^. These distributed circuits interlink neurons in sensory and motor systems also relevant for perceptual and action-execution mechanisms by way of neurons in multimodal areas^33,47,48^. The distributed nature of these ‘action perception circuits’ makes it necessary to use cortical long-distance connections for linking together the motor, acoustic, visual and other perceptual knowledge of engrams and connect them with those parts of the distributed circuits most relevant for memory storage. One of the long-distance connections of the human brain especially important for interlinking action to visual and acoustic information is the arcuate fascicle, AF, which connects frontal premotor and prefrontal with temporal visual, auditory and multimodal areas^17,18,49^. If, as our results suggest, sound symbolic knowledge is based on the co-storage of visual and acoustic information along with the motor aspects of overt bodily actions, the AF will have a main role in sound symbolic processing. From this theoretical consideration, a range of future predictions follow, including the following two: (1) the strength and development of the AF, which are known to vary across individuals^50,51^, might determine or co-determine and therefore correlate with subjects’ variable abilities to make sound-symbolic judgements, (2) subjects with dysfunction of the AF, due to developmental disorders^52^ or cortical lesions, should show no or much reduced ability to perform on sound-symbolic tasks^53^. Hence, it will be an important task for future research to test these predictions and therefore further assess the theoretical proposal about action-perception circuits a basis of sound symbolism. A third prediction is that animals very similar to humans, but without strongly developed AF, should not show any sound-symbolic effects. The latter finding has recently been reported^16^, thus providing at least some independent evidence for the proposed model.

### Summary

We found that healthy human individuals perform similarly well on sound-symbolic matching of ‘round’ and ‘sharp’ pseudowords and abstract shapes as they are able to match diagrams of motor trajectories to the sounds of these same ‘round’ and ‘sharp’ actions. Likewise, the crossed matching of these two conditions worked equally well. Interestingly, there was a significant correlation between our subjects’ performance on sound symbolic and action matching tasks, and this correlation exceeded the level of the relevant control tasks. In addition, similar correlations emerged across sound symbolic, action and crossed conditions, but were absent when comparing performance on the latter and on control tasks. These results indicate common mechanisms of sound-symbolic and action matching and offer an explanation of the hitherto not well-understood iconic link between pseudowords and abstract forms. Although previous models attempted at an explanation based on speech sound production and the presumed shapes of articulatory gestures^24^, closer examination shows that this type of account is insufficient. The novel explanation of sound symbolism based on physical stimulus similarities to the sounds and shapes of bodily actions offers perspectives on modelling the relevant mechanism in a neurobiological framework. Most excitingly, this model offers a biological framework for understanding one type of semantic knowledge, which has long been proposed to lie at the heart of humans’ ability to acquire language and interlink abstract symbols with abstract meanings.

In essence, the present study reports behavioral evidence for a role of action knowledge in explaining sound symbolic congruencies. Our findings are of vital importance from anthropological, linguistic and neurobiological perspectives, as they (1) offer a plausible mechanism behind sound symbolic congruencies relying on the human brain’s action-perception networks and (2) show how body-environment interaction could have contributed to the generation of semantic vocal iconic signals carrying abstract meaning.

## Supplementary information

Supplementary Information.
