# Supplementary material for: Action sound–shape congruencies explain sound symbolism
Source: Sci Rep. 2020 Jul 29;10:12706. doi: 10.1038/s41598-020-69528-4 (PMC7392762; doi:10.1038/s41598-020-69528-4)
Supplement: Supplementary file 1 — Supplementary Information. [file 41598_2020_69528_MOESM1_ESM.pdf]

Action sound-shape congruencies explain sound symbolism

Konstantina Margiotoudi 1,2* and Friedemann Pulvermüller1-4
1Brain Language Laboratory, Department of Philosophy & Humanities, WE4, Freie Universit
at Berlin, 14195 Berlin,Germany
2Berlin School of Mind and Brain, Humboldt Universitat zu Berlin, 10099 Berlin, Germany
3Cluster of Excellence ”Matters of Activity”, Humboldt Universit at zu Berlin, 10099 Berlin, Germany
4EinsteinCenter for Neurosciences,Berlin 10117,Berlin, Germany

Corresponding author: konstantina.margiotoudi@fu-berlin.de

Supplementary Material

TableS1: Mean ratings (M) and standard deviations (SD) obtained from a a Likert scale (1-totally sharp; 7- totally round) for **a)** each action shape and for the sounds produced while drawing these shapes **b)** for each abstract sound symbolic shape **c)** and for the two pseudoword categories (‘sharp’ and ‘round’). All ratings were administrated online.

a)

**Action shapes**	**Shape ratings (N=13)**	**Sound ratings (N=41)**
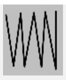	1.07±0.26	2.19±0.98
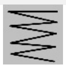	1.15±0.36	2.09±1.04
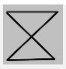	1±0	2.24±1.09
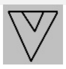	1.15±0.36	2.51±0.86
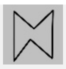	2.15±1.79	1.87±0.81
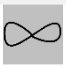	6.69±0.46	5.60±1.33
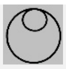	6.76±0.42	5.65±1.45
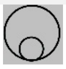	6.53±0.63	4.80±1.69
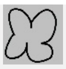	5.92±0.91	4.85±1.31

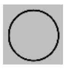	6.78±0.80	5.25±1.60

b)

Sound symbolic shapes	Shape ratings (N=110)
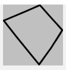	2.36±1.18
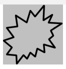	2.31±1.53
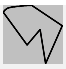	2.26±0.99
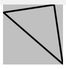	1.58±1.01
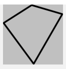	1.80±1.05
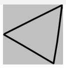	1.62±0.93
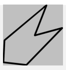	1.85±0.97
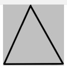	1.49±1.04
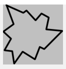	2.34±0.99
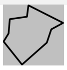	2.40±1.14
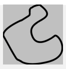	4.73±1.27
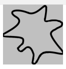	4.79±1.15
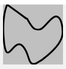	4.75±1.18
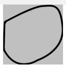	4.93±1.18
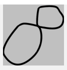	5.22±1.25
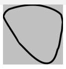	5.68±1.00
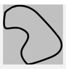	5.01±1.23
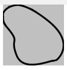	6.09±1.00
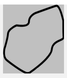	5.49±1.05

c)

Sharp	Round
kiki	nono
keke	nunu
sisi	momo
sese	mumu
fifi	lolo
fefe	lulu
zizi	dodo
zeze	dudu
pipi	gogo
pepe	gugu
**(N=92) M=2.8±0.22**	**M=5.4±0.34**

Table S2. One-tailed Steiger's z test (Steiger, 1980) was used to compare Spearman's correlation coefficients using the package cocor in R (Diedenhofen,2016). Correlation pairs are depicted between the SoSy, Action and the Crossed conditions against the control Animal task. Steiger's Z scores are shown in the middle and p-values in the right column. P-values in bold were significant after controlling for multiple comparisons testing with Bonferroni correction (adjusted threshold p=0.05/12=0.004).

Correlation pairs	Steiger's Z	p-value
**SoSyAction vs. SoSyAnimals**	1.67	0.04
**SosyAction vs. ActionAnimals**	0.55	0.29
**SoSyCrossed1 vs. Crossed1Animals**	3.38	**0.004**
**SoSyCrossed1 vs. SoSyAnimals**	2.62	**0.004**
**ActionCrossed1 vs. ActionAnimals**	0.84	0.19
**ActionCrossed1 vs. Crossed1Animals**	2.74	**0.003**
**SoSyCrossed2 vs. SoSyAnimals**	1.94	0.02
**SoSyCrossed2 vs. Crossed2Animals**	0.92	0.17

**Crossed1Crossed2 vs.Crossed1Animals**	2.37	0.008
**Crossed1Crossed2 vs. Crossed2Animals**	0.69	0.24
**ActionCrossed2 vs. ActionAnimals**	3.40	**0.0003**
**ActionCrossed2 vs. Crossed2Animals**	3.6	**0.0002**
